# Health-care investments for the urban populations, Bangladesh and India

**DOI:** 10.2471/BLT.19.234252

**Published:** 2019-10-21

**Authors:** Daphne CN Wu, Eduardo P Banzon, Hellen Gelband, Brian Chin, Varsha Malhotra, Sonalini Khetrapal, David Watkins, Sungsup Ra, Dean T Jamison, Prabhat Jha

**Affiliations:** a Centre for Global Health Research, Dalla Lana School of Public Health, St Michael’s Hospital & University of Toronto, 30 Bond Street, Toronto, Ontario M5B 1W8, Canada.; b Asian Development Bank, Manila, Philippines.; c Department of Medicine, University of Washington, Seattle, United States of America (USA).; d Institute for Global Health Sciences, University of California, San Francisco, USA.

## Abstract

**Objective:**

To estimate the costs and mortality reductions of a package of essential health interventions for urban populations in Bangladesh and India.

**Methods:**

We used population data from the countries’ censuses and United Nations Population Division. For causes of mortality in India, we used the Indian Million Death Study. We obtained cost estimates of each intervention from the third edition of *Disease control* *priorities*. For estimating the mortality reductions expected with the package, we used the *Disease control priorities* model. We calculated the benefit–cost ratio for investing in the package, using an analysis based on the Copenhagen Consensus method.

**Findings:**

Per urban inhabitant, total costs for the package would be 75.1 United States dollars (US$) in Bangladesh and US$ 105.0 in India. Of this, prevention and treatment of noncommunicable diseases account for US$ 36.5 in Bangladesh and U$ 51.7 in India. The incremental cost per urban inhabitant for all interventions would be US$ 50 in Bangladesh and US$ 75 in India. In 2030, the averted deaths among people younger than 70 years would constitute 30.5% (1027/3362) and 21.2% (828/3913) of the estimated baseline deaths in Bangladesh and India, respectively. The health benefits of investing in the package would return US$ 1.2 per dollar spent in Bangladesh and US$ 1.8 per dollar spent in India.

**Conclusion:**

Investing in the package of essential health interventions, which address health-care needs of the growing urban population in Bangladesh and India, seems beneficial and could help the countries to achieve their 2030 sustainable development goals.

## Résumé

**Objectif:**

Estimer les coûts et les réductions de la mortalité liés à un ensemble d'interventions essentielles en santé pour les populations urbaines au Bangladesh et en Inde.

**Méthodes:**

Nous avons utilisé des données démographiques provenant des recensements des pays et de la Division de la population des Nations Unies. Concernant les causes de la mortalité en Inde, nous avons utilisé un document intitulé « Indian Million Death Study ». Nous avons obtenu des estimations du coût de chaque intervention à partir de la troisième édition du rapport « *Disease control priorities* ». Pour estimer les réductions de la mortalité attendues grâce à l'ensemble des interventions, nous avons utilisé le modèle du projet *Disease control priorities*. Nous avons calculé le rapport avantages-coûts de l'investissement dans cet ensemble d'interventions à l'aide d'une analyse fondée sur la méthode du Consensus de Copenhague.

**Résultats:**

Les coûts totaux par habitant en milieu urbain pour l'ensemble des interventions seraient de 75,1 dollars des États-Unis (US$) au Bangladesh et 105,0 US$ en Inde. Sur ces sommes, la prévention et le traitement des maladies non transmissibles représentent 36,5 US$ au Bangladesh et 51,7 US$ en Inde. Le surcoût par habitant en milieu urbain pour toutes les interventions serait de 50 US$ au Bangladesh et 75 US$ en Inde. En 2030, les décès évités chez les personnes âgées de moins de 70 ans représenteraient 30,5% (1027/3362) et 21,2% (828/3913) du nombre de décès estimé de référence au Bangladesh et en Inde, respectivement. Les effets bénéfiques pour la santé de l'investissement dans l'ensemble des interventions permettraient un rendement de 1,2 US$ par dollar dépensé au Bangladesh et 1,8 US$ par dollar dépensé en Inde.

**Conclusion:**

L'investissement dans l'ensemble d'interventions essentielles en santé, qui répond aux besoins en soins de santé de la population urbaine croissante au Bangladesh et en Inde, semble bénéfique et pourrait aider les pays à atteindre leurs objectifs de développement durable à l'horizon 2030.****

## Resumen

**Objetivo:**

Estimar los costos y las reducciones de la mortalidad de un conjunto de intervenciones sanitarias esenciales para las poblaciones urbanas de Bangladesh y la India.

**Métodos:**

Se utilizaron datos de población de los censos de los países y de la División de Población de las Naciones Unidas. Para las causas de mortalidad en la India, se utilizó el Indian Million Death Study (Estudio del Millón de Muertes en la India). Se obtuvieron estimaciones de costos de cada intervención a partir de la tercera edición de *Disease control* *priorities *(Prioridad para el control de enfermedades). Para estimar las reducciones de mortalidad esperadas con el paquete, se utilizó el modelo de *Disease control priorities*. Se calculó la relación costo-beneficio para invertir en el paquete, utilizando un análisis basado en el método del Consenso de Copenhague.

**Resultados:**

Por habitante urbano, el costo total del paquete sería de 75,1 dólares estadounidenses (USD) en Bangladesh y 105,0 USD en la India. La prevención y el tratamiento de las enfermedades no transmisibles representan 36,5 USD en Bangladesh y 51,7 USD en la India. El costo adicional por habitante urbano para todas las intervenciones sería de 50 USD en Bangladesh y 75 USD en la India. En 2030, las muertes que se evitarían entre las personas menores de 70 años representarían el 30,5 % (1 027/3 362) y el 21,2 % (828/3 913) de las muertes estimadas de referencia en Bangladesh y la India, respectivamente. Los beneficios para la salud de invertir en el paquete devolverían 1,2 USD por cada dólar gastado en Bangladesh y 1,8 USD por cada dólar gastado en la India.

**Conclusión:**

La inversión en el conjunto de intervenciones sanitarias esenciales, que abordan las necesidades de atención de la salud de la creciente población urbana de Bangladesh y la India, parece beneficiosa y podría ayudar a los países a alcanzar sus objetivos de desarrollo sostenible para 2030.

## ملخص

**الغرض:**

تقدير التكاليف وحالات انخفاض معدلات الوفيات لمجموعة من التدخلات الصحية الأساسية لسكان الحضر في بنغلاديش والهند.

**الطريقة:**

قمنا باستخدام البيانات السكانية الصادرة عن تعدادات البلدان وقسم السكان بالأمم المتحدة. وبالنسبة لأسباب الوفيات في الهند، قمنا باستخدام "دراسة وفاة المليون الهندي". حصلنا على تقديرات لتكلفة كل تدخل من الطبعة الثالثة من أولويات *مكافحة الأمراض *. ولتقدير حالات انخفاض الوفيات المتوقعة مع الحزمة، قمنا باستخدام نموذج *أولويات مكافحة الأمراض *. قمنا باحتساب نسبة الفائدة والتكلفة للاستثمار في الحزمة، باستخدام تحليل يعتمد على طريقة إجماع كوبنهاجن.

**النتائج:**

سيكون إجمالي تكاليف الحزمة لكل مقيم بالحضر هو 75.1 دولاراً أمريكياً (دولار أمريكي) في بنغلاديش، و105.0 دولاراً أمريكياً في الهند. ومن هذه المبالغ، كانت الوقاية من الأمراض غير المعدية وعلاجها تمثل 36.5 دولاراً أمريكياً في بنغلاديش، و51.7 دولاراً أمريكياً في الهند. ومن هذه المبالغ، كانت الوقاية من الأمراض غير المعدية وعلاجها تمثل 36.5 دولاراً أمريكياً في بنغلاديش، و51.7 دولاراً أمريكياً في الهند. بحلول عام 2030، فإن نسبة حالات الوفيات التي تم تجنبها بين الأشخاص الأصغر من 70 عاماً سوف تصل 30.5% (1027/3362)، و21.2% (828/3913) من وفيات خط الأساس التقديري في بنغلاديش والهند، على الترتيب. ستعود الفوائد الصحية للاستثمار في الحزمة بمبلغ 1.2 دولار أمريكي لكل دولار تنفقه بنغلاديش، و1.8 دولار أمريكي لكل دولار تنفقه الهند.

**الاستنتاج:**

إن الاستثمار في حزمة التدخلات الصحية الضرورية، والتي تلبي احتياجات الرعاية الصحية لنسبة السكان الحضرية المتنامية في بنغلاديش والهند، يبدو مفيدًا ويمكن أن يساعد البلدان على تحقيق أهداف التنمية المستدامة لعام 2030.

## 摘要

**目的:**

旨在评估孟加拉国和印度城市人口一系列基本卫生干预措施的成本和死亡率降低情况。

**方法:**

我们使用了来自各国人口普查和联合国人口司的人口数据。我们使用印度百万死亡研究中的数据调查印度人口死亡原因。我们从第三版《*疾病控制》*优先事项中获得了每项干预措施的成本估算数据。我们使用了*疾病控制优先事项*模型估计该方案预期下降的死亡率。我们使用基于哥本哈根共识方法的分析，计算了投资该系列计划的效益成本比。

**结果:**

每位孟加拉国城市居民的总费用为 75.1 美元 (US$)，每位印度城市居民的总费用为 105.0 美元。其中，每位孟加拉国城市居民用于预防和治疗非传染性疾病的费用为 36.5 美元，印度为 51.7 美元。孟加拉国所有干预措施的城市居民人均增量成本为 50 美元，印度为 75 美元。到 2030 年，70 岁以下人群的避免死亡人数将分别占孟加拉国和印度估计基线死亡人数的 30.5% (1027/3362) 和 21.2% (828/3913)。投资该系列计划的卫生保健效益将使孟加拉国和印度分别获得人均 1.2 美元和 1.8 美元的回报。

**结论:**

投资该系列基本卫生干预措施可解决孟加拉国和印度不断增长的城市人口对卫生保健的需求，有益于帮助这些国家实现其 2030 年可持续发展目标。****

## Резюме

**Цель:**

Оценить затраты и снижение уровня смертности в результате проведения комплекса основополагающих интервенций в области здравоохранения среди городского населения Бангладеш и Индии.

**Методы:**

Авторы использовали данные переписи населения в этих странах и данные, предоставленные отделом народонаселения Организации Объединенных Наций. Данные о причинах смертности в Индии были взяты из индийского исследования Million Death Study (Миллион смертей). Примерную оценку затрат на соответствующее вмешательство авторы получили на основании третьего издания приоритетов «*Контроль заболеваний*». Для оценки ожидаемого снижения уровня смертности в результате применения рассматриваемого комплекса мер была использована модель *приоритетов контроля заболеваний.* Авторы рассчитали соотношение «затраты-выгода» для инвестиций в комплекс мер, пользуясь анализом, основанным на методе Копенгагенского консенсуса.

**Результаты:**

Общие затраты на внедрение комплекса мер из расчета на одного горожанина составили 75,1 доллара США в Бангладеш и 105,0 доллара США в Индии. Из этой суммы на профилактику и лечение неинфекционных заболеваний было потрачено 36,5 доллара США в Бангладеш и 51,7 доллара США в Индии. Рост затрат из расчета на душу городского населения для всех вмешательств составил 50 долларов США в Бангладеш и 75 долларов США в Индии. В 2030 году уровень предотвращенной смертности среди людей моложе 70 лет составит около 30,5% (1027 из 3362 случаев) и 21,2% (828 из 3913 случаев) от предполагаемого базового уровня смертности в Бангладеш и Индии соответственно. Окупаемость инвестиций в рассматриваемом комплексе мер составит 1,2 доллара США для Бангладеш и 1,8 доллара США в Индии на каждый вложенный доллар США.

**Вывод:**

Инвестиции в комплекс основополагающих мер в сфере здравоохранения, направленных на удовлетворение потребностей в медицинском обслуживании растущего городского населения Бангладеш и Индии, представляются благотворными и могут помочь этим странам достичь целей устойчивого развития, запланированных на 2030 год.****

## Introduction

Cities promote national economic growth and prosperity, innovation and overall national welfare. The United Nations (UN) has pointed out that modern cities exhibit contrasts between wealth and poverty, opportunity and deprivation and vibrant potential and systemic decay.^1^ Cities have natural advantages in providing all kinds of services, not least because they are national economic drivers, with access to proportionately greater financing mechanisms than rural areas. Their size allows for a greater variety of services and economies of scale compared with sparsely populated areas. However, they also face challenges, for example, in establishing new health facilities where real estate is expensive and scarce, and in incorporating the long-neglected urban poor into comprehensive planning.^2^

According to the UN, the proportion of the world’s population living in urban areas will increase, from an estimated 55% in 2018 to an estimated 68% by 2050.^3^ Bangladesh and India are experiencing some of the highest urban population growth rates in the world. The UN projects that the urban population will grow from 48 million in 2011 to 84 million in 2030 in Bangladesh. In India, the projected population increase is from 377 million to 612 million.^3^ This increase will be largely due to internal migration and natural population growth. Slums will account for an ever-greater proportion of urban inhabitants. In 2015, 62 cities in Bangladesh and India had more than 1 million population and five had more than 10 million; by 2030, 77 cities will exceed a population of 1 million and eight will exceed 10 million.^3^

To meet the health needs of the growing urban population, health-care services need to expand. Currently, both countries have a mix of public and private health-care provision. In India, publicly-financed health services have been provided exclusively by public sector facilities, with little formal attention to either regulating the private sector or to delivering publicly-financed services through private providers.^4^ The Bangladeshi government has for the past two decades used some public financing to fund services through the nongovernmental organizations. In both countries, health infrastructure and services have steadily improved, but are still inadequate to serve the population need. Scarce efforts to improve urban health have been made over the last several decades, either by national governments or external partners.^5^^,^^6^

To assess the cost and benefit of providing interventions for major public health, prevention and treatment needs for populations in Bangladesh and India, we identified interventions from the nine volumes of the third edition of the *Disease control priorities.*^7^ The 208 interventions identified constitute a package of essential health services covering the most common causes of visits to doctors and admission to hospitals during the life course. This package includes almost all of the 218 interventions included in *Disease control priorities,* omitting only those that are not relevant to South Asian populations, e.g. prevention and treatment of African trypanosomiasis. Box 1 presents some examples of the interventions; the full list is available in the data repository.^8^ Here we estimate the costs, mortality reduction and the benefit–cost ratio of providing this package for the urban populations in Bangladesh and India.

Box 1 **Examples of interventions included in the suggested urban package of essential health services for Bangladesh and India**

**Maternal, perinatal and childhood conditions**

• Management of labour and delivery in low-risk women by skilled attendants, including basic neonatal resuscitation following delivery, and in high-risk women, including operative delivery.

• Childhood immunizations, including DPT, polio, BCG, measles, hepatitis B, *Haemophilus influenzae* type B and rubella vaccines.

**Infectious diseases**

• Active case finding of high-risk individuals (e.g. people living with HIV) with tuberculosis symptoms and linkage to care.

• In all malaria-endemic areas, diagnosis with rapid test or microscopy followed by treatment with artemisinin-based combination therapy (or current first-line combination). Where rapid test and microscopy are unavailable, patients with febrile illness receive presumptive treatment with artemisinin-based combination therapy and patients with severe illness receive in addition antibiotics.

**Noncommunicable diseases (such as cardiovascular disease, cancer, mental health, rehabilitation and palliative care)**

• Substantial increases in the excise taxes on manufactured cigarettes.

• Opportunistic screening for hypertension for all adults and initiation of treatment among individuals with severe hypertension and/or multiple risk factors.

• Long term management of ischaemic heart disease, stroke and peripheral vascular disease with aspirin, β blockers, blood pressure lowering pills, and statins (as indicated) to reduce risk of further events.

• Management of acute exacerbations of asthma and COPD using systemic steroids, inhaled β-agonists, and if indicated, oral antibiotics and oxygen therapy.

• Early detection and treatment of early-stage breast, cervical, breast, and childhood cancers.

• Management of depression and anxiety disorders with psychological and generic antidepressant therapy.

• Rehabilitation programmes for cardiac and pulmonary conditions.

• Essential palliative care and pain control measures, including oral immediate release morphine, and medicines for associated symptoms.

**Injuries**

• Trauma related surgical procedures, such as laparotomy and amputations.

• Rehabilitation for patients following acute injury or illness.

• Gender-based violence care, including counselling, provision of emergency contraception and rape-response referral.

BCG: bacillus Calmette–Guérin; COPD: chronic obstructive pulmonary disease; DPT: diphtheria, pertussis and tetanus; HIV: human immunodeficiency virus.Note: All 208 interventions included in the package are available from the data repository.^8^

## Methods

### Demography and disease burden

For both countries, we used population data from the 1991, 2001 and 2011 censuses,^9^^,^^10^ and the UN Population Division’s (UNPD’s) population projection for the year 2030.^3^ For cause-specific mortality estimates in urban India, we used data from the Medical Certification of Cause of Death for the years before 2001,^11^ and from the Million Death Study for 2001 and onward.^12^ Cause-specific mortality data for all ages are not available for Bangladesh.

### Cost of the package

To estimate the cost of the package, we obtained cost estimates of each intervention for lower-middle income countries in 2012 United States dollars (US$), from the third edition of *Disease control priorities*.^7^^,^^13^^–^^20^ We used 2012 exchange rates from the World Development Indicators to convert costs into Bangladeshi Taka and Indian Rupees, the World Development Indicators consumer price index to convert to 2016 local costs, and finally converted back to US$ using the 2016 exchange rate.^21^ We based costs on a population of one million and an 80% coverage level for all included interventions. We calculated two cost estimates of the package, incremental and total costs. Incremental cost, that is, the additional cost that would be needed to provide 80% population coverage, was calculated as:
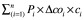
(1)where *P_i_* is the population in need of the intervention *i*, Δ*co_i_* is the additional proportion of individuals that is needed to reach 80% coverage (that is, 80% minus current coverage level), *c_i_* is the yearly cost per person of intervention *i*.

The total cost for 80% coverage, that is the total costs of current spending plus incremental cost, was calculated as:
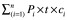
(2)where *t* is the target coverage level of 80%.^22^

To estimate the population in need of an intervention, we used national surveys, ministry reports and population-based registries to obtain incidence or prevalence data of relevant conditions. For various reproductive, maternal and child conditions, and gender-based violence, we used urban-specific data from the India National Family Health Survey,^23^ Bangladesh Demographic and Health Survey,^24^ and Bangladesh Report on Violence Against Women Survey.^25^ For cancer incidence, we used 2012 data from the International Agency for Research on Cancer GLOBOCAN database for Bangladesh^26^ and data for urban India (2012–2014) from the National Population-Based Cancer Registry.^27^ For conditions where incidence and prevalence data were not reported by urban and rural sectors, we used national estimates from published literature, government reports, World Health Organization (WHO) reports and the Global Burden of Disease model-based estimates for South-Asia for 2016.^28^ The earliest data were from 2011. Where epidemiological data were not available, we used estimates from the third edition of *Disease control priorities* for lower middle-income countries.^22^

For baseline coverage data, we used the Indian National Family Health Survey and the Demographic Health Survey in Bangladesh, which provide data on the urban population for most reproductive, maternal and child health, and household sanitation interventions.^23^^,^^24^ For other interventions, we used data from the published literature. For interventions similar to an intervention with available coverage data, we used that intervention as a proxy. We used WHO coverage estimates for malaria and tuberculosis diagnosis and treatment.^29^^,^^30^ For missing data (e.g. for mental health disorders) we used baseline coverage estimates for lower middle-income countries from the third edition of *Disease control priorities*.^7^^,^^22^

To account for the costs of infrastructure, surveillance, regulation and other support activities, we added 40% of the total direct cost, that is, personnel, drugs and equipment costs. This infrastructure excess was based on an earlier detailed costing analysis for India.^4^ Using the above inputs, we estimated overall current annual spending, current annual spending per capita, and the incremental and total annual cost needed to achieve 80% coverage of the package. We also allocated the cost across major disease groups, and by platforms of health system (that is, population-based interventions, community services, health centres, first-level hospitals and referral and specialized hospitals) and the type of care provided (that is, urgent, recurrent for chronic diseases and others, such as childhood immunization).

### Mortality reduction

We estimated the number of premature deaths (before 70 years of age) averted by the package by first estimating the age and sex distributions for urban population in Bangladesh and India for 2030. We did so by applying the age and sex distributions of the urban population of the 2011 Bangladesh and India censuses to the UNPD urban population projection for 2030.^3^^,^^9^^,^^10^ We projected 2030 baseline deaths using cause-specific mortality rates from the Million Death Study for urban India.^12^ Such data were unavailable for Bangladesh, so we used the average cause-specific mortality rates in urban West Bengal and Assam, the major Indian states bordering Bangladesh, as proxies. We used the effect sizes of the package interventions on mortality reduction for lower-middle income countries from a published working paper,^31^ assuming uniform effect sizes across all age groups and 80% efficiency in intervention delivery at baseline. We compared the estimated mortality reduction to the so-called 40x30 reduction target, which is a set of selected disease-specific targets to help achieve the sustainable development goal 3.^32^ This reduction target aims for a 40% reduction in deaths among people younger than 70 years; a two-third reduction in child and maternal mortality and mortality due to human immunodeficiency virus infection, tuberculosis and malaria; and one-third reduction in premature deaths from other communicable diseases, injuries and noncommunicable diseases.^32^

### Benefit–cost ratio

To estimate the benefit–cost ratio for investing in the package, we used a published method^33^ that is based on the Copenhagen Consensus method.^34^ We converted the number of deaths averted in the age groups 0–4 years and 5–69 years to disability-adjusted life years (DALYs). For the age group 0–4 years we used a factor of 97 DALYs per death averted. For the age group 5–69 years, we used 97 DALYs per death averted for the ages 5–49 years and 42 DALYs per death averted for ages 50–69 years. The conversion factors were derived by dividing the total all-cause DALYs by the total number of deaths in each age group in lower-middle income countries from the 2016 WHO global health estimates.^35^ We monetized the DALYs conservatively by multiplying by twice the 2016 gross domestic product (GDP) per capita in each country. We obtained GDP per capita from the World Development Indicators.^21^ We applied a 3% discount rate to costs and benefits over 15 years.

### Sensitivity analysis

To examine the effect of the package on mortality reduction at different levels of delivery efficiency and coverage levels, we conducted sensitivity analyses at 70%, 80%, 90% and 95% efficiency in intervention delivery and at 60%, 70%, 80%, 90% and 100% coverage levels. All analyses were performed in Stata version 15.1 (StataCorp. LCC, College Station, United States of America).

## Results

### Demographics in 2030

As urban populations are increasing over the next decades, the population structure will shift towards middle and older ages, with the largest increases in the 30–69-year age group in both countries. The proportion of population aged 30–49 years will increase from 26.7% (12.8 million/48.1 million) in 2011 to 32.0% (26.9 million/84.1 million) in 2030 in urban Bangladesh, and from 28.2% (106.2 million /377.1 million) to 31.6% (193.5 million /611.5 million) in urban India. The proportion of population aged 50–69 years will increase from 10.4% (5.0 million /48.1 million) to 17.2% (14.5 million /84.1 million) in urban Bangladesh, and from 13.1% (49.2 million /377.1 million) to 18.2% (111.4 million /611.5 million) in urban India (available in the data repository).^8^ This shift reflects migration patterns, a progress in preventing deaths in infancy and childhood, natural population growth and increased life expectancy due to income growth, education and better health-care services.^4^

### Causes of mortality

**Fig. 1:**
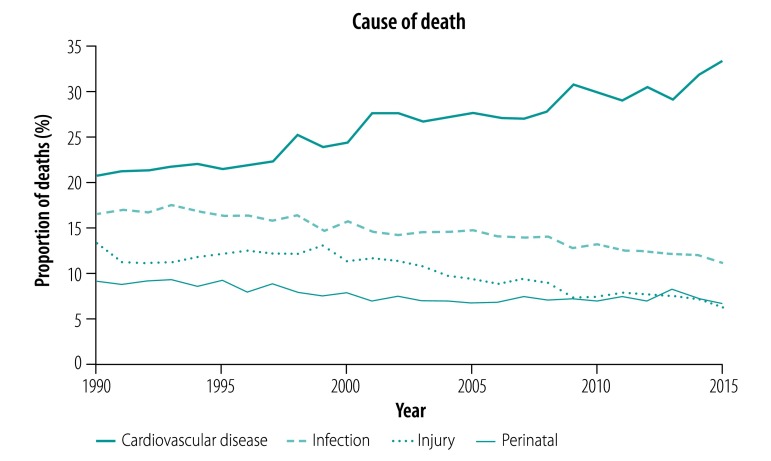
Distribution of leading causes of mortality in urban India, 1990–2015

In urban India, noncommunicable disease deaths are rising as a proportion of overall mortality, such as cardiovascular disease, respiratory diseases and injuries (Fig. 1 and data repository).^8^ However, infectious diseases are still a problem. Urban crowding, lack of clean water and sanitation and mobility contribute to continuing transmission of infectious diseases. For example, India has a high tuberculosis burden, and Mumbai, a megacity of more than 18 million people, is a particular hotspot.^36^

### The intervention package

#### Cost

Table 1 shows the current annual spending on health services for the urban population in Bangladesh and India, and the incremental and the total annual cost if interventions in the package were implemented. At 80% coverage, the suggested package would cost US$ 75.1 per urban inhabitant in Bangladesh and US$ 105.0 in India by 2030. To achieve this coverage, the Bangladeshi government would need to increase their health spending by US$ 49.3 per urban inhabitant, while the Indian government would need to increase this sum by US$ 74.6 per urban inhabitant. To cover 80% of urban populations in 2030, both Bangladesh and India would need to spend about an additional 2.0% of the 2016 GDPs. This spending is an addition to the current spending of about 1.0% of the 2016 GDP.

**Table 1:** Estimated cost of an urban package of essential health services for a city with a population of one million, Bangladesh and India

Variable	Cost per urban inhabitant, US$^a^			% of service delivery costs^b^
	Current annual spending	Incremental annual cost	Total annual cost in 2030	
**Bangladesh^c^**				
Package				
Age-related^d^	6.5	3.2	9.7	18.1
Infectious disease	6.5	5.4	11.9	22.2
Noncommunicable disease and injury	6.5	30.0	36.5	68.1
Health service	3.5	4.4	8.0	14.8
Cost after removing duplicated interventions^e^	18.4	35.2	53.6	100.0
Health system cost^f^	7.4	14.1	21.5	NA
Total cost	25.8	49.3	75.1	NA
Total cost to cover all urban population by 2030, % of national GDP in 2016^g^	1.0	1.9	2.9	NA
**India^h^**				
Package				
Age-related^d^	7.3	1.5	8.9	11.8
Infectious diseases	7.9	10.9	18.9	25.1
Noncommunicable disease and injury	8.7	43.0	51.7	68.9
Health services	3.2	6.1	9.3	12.5
Cost after removing duplicated interventions^e^	21.7	53.3	75.0	100.0
Health system cost^f^	8.7	21.3	30.0	NA
Total cost	30.4	74.6	105.0	NA
Total cost to cover all urban population by 2030, % of national GDP in 2016^i^	0.8	2.0	2.8	NA

GDP: gross domestic product; NA: not applicable; US$: United States dollars.

^a^ Values are US$ except values for total cost to cover all urban population by 2030 that are in percentage.

^b^ Some interventions are included in more than one package, hence the sum of the percentages is larger than 100.

^c^ Total urban population of 2016 was 55.4 million people in 2016 and is projected to be 84.1 million in 2030.

^d^ Age-related package includes maternal and newborn health interventions, child health interventions, school-age health and development interventions, adolescent health and development interventions, and reproductive health and contraception interventions.

^e^ Some of the interventions are included in more than one package.

^f^ Calculated as 40% of total service delivery cost.

^g^ Based on GDP per capita of US$ 1358.78.

^h^ Total urban population of 2016 was 439.5 million people and is projected to be 611.5 million people.

^i^ Based on GDP per capita of US$ 1717.47.

Notes: We assumed that the interventions would cover 80% of the people. The monetary values are in 2016 US$.

**Fig. 2:**
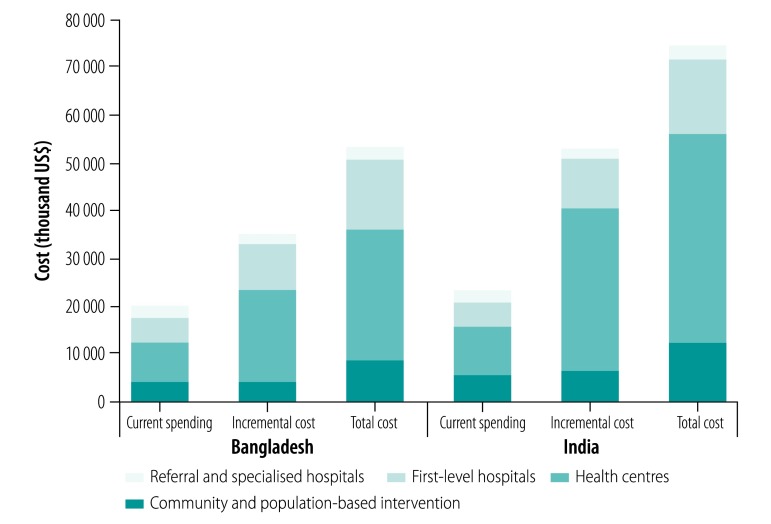
Projected cost for an urban package of essential health services in a city with a population of one million, by type of platform of intervention delivery, Bangladesh and India, 2030

**Fig. 3:**
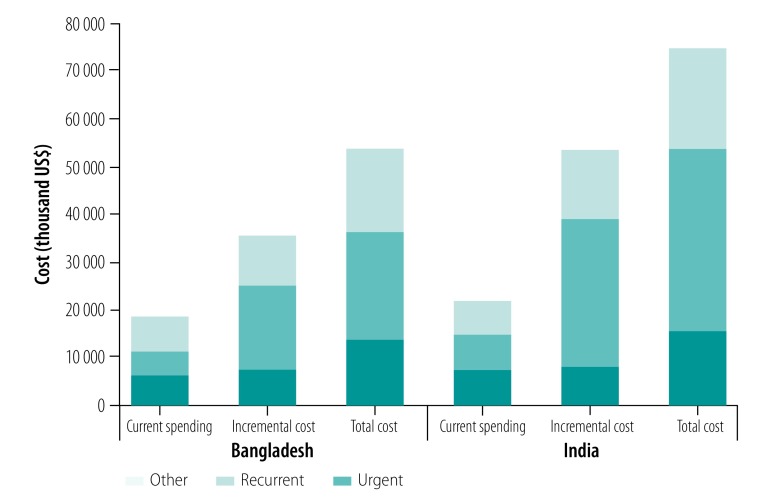
Projected cost of an urban package of essential health services, in a city with a population of one million, by type of intervention, Bangladesh and India, 2030

Note: Recurrent provision includes interventions for chronic conditions. Other type of provision is interventions that are neither urgent nor recurrent, but nonetheless are important, such as childhood immunizations and provision of iron and folic acid supplementation for pregnant women.

The total incremental cost would be US$ 35.2 million in Bangladesh and US$ 53.3 million in India (Table 1). In both countries, most of the incremental cost would be invested in health centres, followed by first-level hospitals and community- and population-based interventions. Investment in referral and specialized hospitals accounts for only 5.3% (US$ 1.9 million) of the total incremental cost in Bangladesh and 3.8% (US$ 2.0 million) in India (Fig. 2). Examining the distribution of package costs by type of provision showed that in both countries, more than half of the incremental cost, 50.9% (US$ 17.9 million) in Bangladesh and 58.6% (US$ 31.2 million) in India, would be invested in management of chronic conditions to reduce risk of further events. Routine interventions would account for 28.2% (9.9 million) of incremental costs in Bangladesh and 26.5% (14.1 million) in India. Urgent conditions account for the remaining incremental costs (Fig. 3).

US$: United States dollars

#### Mortality reduction

If the countries’ governments would implement the recommended package, we estimate that per million population, the number of premature deaths in the year 2030 would decrease from 3362 to 2335 in Bangladesh and from 3913 to 3085 in India. These averted deaths would constitute 30.5% and 21.2% of the baseline deaths in Bangladesh and India, respectively. The package would contribute to achieving the 40x30 reduction target by averting 76.3% (1027/1345 per million population) of premature deaths set by the target in Bangladesh. In India, this percentage would be 52.9% (828/1565 per million population). Furthermore, both countries would make progress towards the 40x30 target for under-five mortality by averting 168 deaths in Bangladesh and 163 deaths in India per million urban population, resulting in 58.2% of deaths set by the 40x30 reduction target to be averted (Table 2). Progress towards the 40x30 target for noncommunicable diseases would be much higher in Bangladesh (90.9%; 586/645) than in India (64.5%; 504/782).

**Table 2:** Estimated deaths averted by an urban package of essential health services in a city with a population of one million in Bangladesh or India, 2030

Group	Bangladesh					India			
	No. of projected deaths	No. of deaths averted if 40x30 target achieved^a^	Package implemented			No. of projected deaths	No. of deaths averted if 40x30 target achieved^a^	Package implemented	
			Deaths averted, no. (% of projected deaths)	% of 40x30 target				Deaths averted, no. (% of projected deaths)	% of 40x30 target
**Age**									
0–4 years	432	288	168 (38.9)	58.2		420	280	163 (38.8)	58.2
5–69 years	2930	1057	859 (29.3)	81.3		3493	1285	665 (19.0)	51.8
0–69 years	3362	1345	1027 (30.5)	76.3		3913	1565	828 (21.2)	52.9
**Cause^b^**									
Infectious, maternal and perinatal conditions	564	318	250 (44.3)	78.5		586	297	132 (22.5)	44.3
Noncommunicable diseases	1935	645	586 (30.3)	90.9		2345	782	504 (21.5)	64.5
Injuries	272	91	23 (8.5)	25.0		432	144	30 (6.9)	20.8

^a^ The 40x30 reduction target aims for a 40% reduction of deaths among individuals 0–69 years, a two-third reduction in child and maternal mortality and mortality due to human immunodeficiency virus infection, tuberculosis and malaria, and one-third reduction in premature deaths from other communicable diseases, injuries and noncommunicable diseases.^32^

^b^ In age group 5–69 years.

Notes: The package consists of 208 health interventions we identified through the third edition of *Disease control priorities*.^7^ Examples of interventions are presented in Box 1 and the full list is available in the data repository.^8^ Implementation of the package would also reduce mortality in people older than 70 years. However, we have not included these benefits.

#### Benefit–cost ratio

We estimated that in Bangladesh, the benefits of investing in the package would yield US$ 1.2 of benefits to each dollar spent; in India, the benefit would be US$ 1.8 (Table 3).

**Table 3:** Benefit-cost-ratio for an urban package of essential health services in a city with a population of one million in Bangladesh or India, 2016

Cost or benefit	Bangladesh	India
Total cost of package, million US$	75	105
GDP per capita, US$	1 359	1 717
Total no. of deaths averted	1 027	828
Total DALYs averted	33 558	55 914
Benefit in monetary terms, million US$	91	192
Discounted cost of package, million US$^a^	48	67
Discounted benefit, million US$^a^	59	123
Benefit-cost-ratio with 3% discounting	1.2	1.8

DALY: disability-adjusted life year; GDP: gross domestic product; US$: United States dollars.

^a^ Cost and benefit discounted at 3% over 15 years

#### Sensitivity analysis

**Fig. 4:**
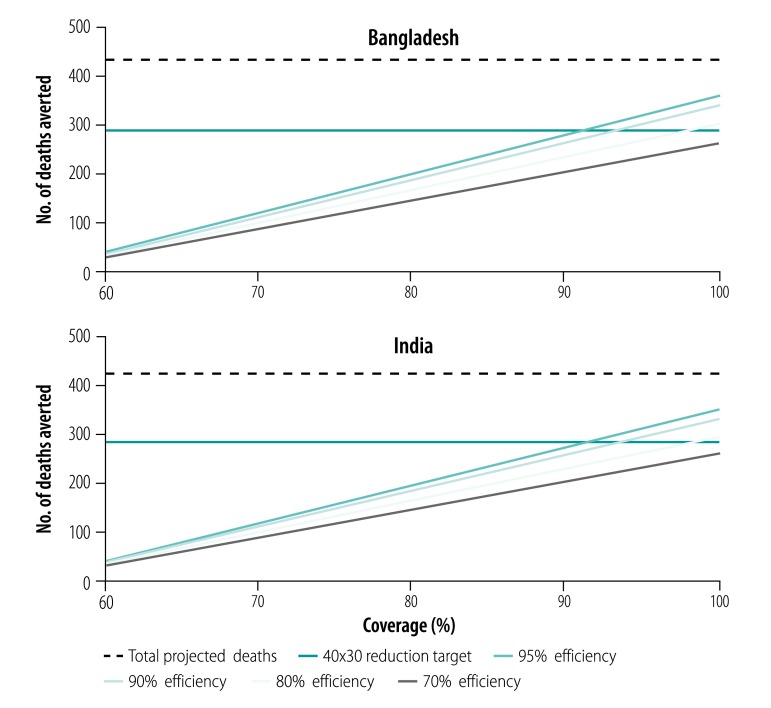
Sensitivity analysis of reduction in under-five mortality for an urban package of health interventions, Bangladesh and India

Notes: The estimations are based on cities of one million population. The 40x30 reduction target aims for a 40% reduction in deaths among people younger than 70 years; a two-third reduction in child and maternal mortality and mortality due to human immunodeficiency virus infection, tuberculosis and malaria; and one-third reduction in premature deaths from other communicable diseases, injuries and noncommunicable diseases.^32^

**Fig. 5:**
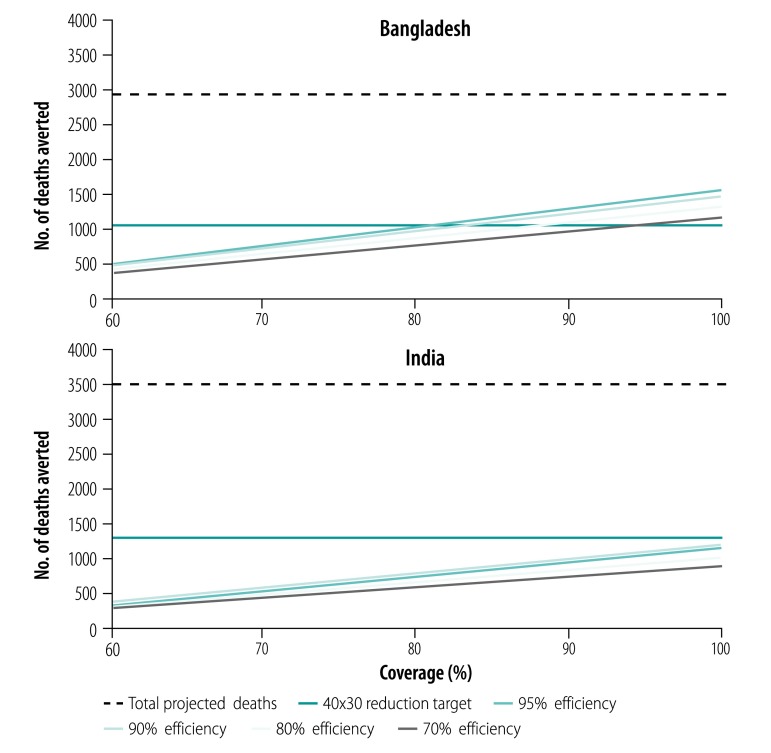
Sensitivity analysis of reduction in mortality among individuals 5–69 years for an urban package of health interventions, Bangladesh and India

Notes: The estimations are based on cities of one million population. The 40x30 reduction target aims for a 40% reduction in deaths among people younger than 70 years; a two-third reduction in child and maternal mortality and mortality due to human immunodeficiency virus infection, tuberculosis and malaria; and one-third reduction in premature deaths from other communicable diseases, injuries and noncommunicable diseases.^32^

In Fig. 4 and Fig. 5, we have plotted varying intervention delivery efficiencies and coverage levels to demonstrate whether they would achieve the 40x30 reduction target for a city of one million population. Both countries could only achieve the 40x30 target for under-five mortality with at least 90% coverage and at least 90% efficiency in intervention delivery. In the 5–69-year age group, we found that in Bangladesh, the 40x30 reduction target could be achieved if the package was implemented with at least 90% efficiency and at least 85% coverage, or at least 80% efficiency and at least 90% coverage. In India, while substantial progress could be made, the 40x30 target could not be achieved even at the highest coverage and efficiency levels tested.

## Discussion

We estimated that investing in a hypothetical package of 208 cost–effective health interventions that addresses the health-care needs of the growing urban population in Bangladesh and India is beneficial. For example, noncommunicable disease burden can be controlled with treatments that are low cost and feasible to deliver in primary care and hospital facilities, coupled with public health measures to reduce the impact of major risk factors, such as smoking and obesity. However, access to many of the most cost–effective health system interventions is currently limited, especially among the poorest population groups. Expanding universal coverage of essential health interventions for adults could have a similar levelling effect as seen for improving child heath with free or inexpensive vaccines and primary care. 

In the last decades, the advance towards universal health coverage (UHC) and the recognition that a healthy population is cost beneficial with substantial welfare gains^33^^,^^34^ make a compelling case for public investment in urban health. In India, public expenditure on health was just over 1.0% of GDP in 2015^37^ and in Bangladesh, this percentage was 0.8% in 2014.^38^ In both countries, out-of-pocket spending on health accounted for more than two-thirds of the total health expenditure.^38^ Increasing public spending on health could reduce out-of-pocket payments, as shown in other countries,^39^ while improving the quality of services.^4^ Increased public health expenditure advances UHC and avoids the impoverishment that often results from out-of-pocket expenditures.^7^

We estimated that to cover all million-plus cities in Bangladesh and India by 2030, governments must increase their current health spending about threefold. While this increase is large, this level of health spending is consistent with WHO recommendations,^40^ India’s Choosing Health Report^4^ and the National Commissions for Macroeconomics and Health in both countries.^41^^,^^42^ Expecting that the governments of Bangladesh or India can immediately increase their expenditures to the suggested levels would be unrealistic, but they can plan for a decade-long scale-up of health spending and deploy new tactics to increase revenue to finance health-care. For example, they could require mandatory contributions from people with high income through taxation, and/or compulsory earmarked contributions for health insurance.^43^ Innovative financing schemes, such as issuing diaspora bonds to expatriates and imposing taxes on foreign exchange transactions, could be adopted.^44^ For instance, in 2017, an average daily turnover of foreign exchange amounted to US$ 58.0 billion in India;^45^ a transaction levy of 0.005% on every working day would yield about US$ 630.0 million a year. Taxes on tobacco and other harmful substances and reduced government subsidies on fossil fuels have also been recommended as strategies to increase revenue available for the health sector. Although tobacco taxes would not themselves provide enough to cover the financial needs of UHC,^46^ they could make significant contributions.^47^

Given the enormous gains in health and welfare from healthy populations in cities, countries could also responsibly take low-interest loans from the international market, with federal guarantees for on-lending or for grants to cities. Borrowing from development partners, such as the Asian Development Bank and the World Bank is also attractive, given the large returns produced by investments in population health. Novel mechanisms to enable cities to borrow or spend with federal financing and support could also be developed. For example, in addition to providing loan financing, the Asian Development Bank provides technical assistance and advisory services to enhance and accelerate operationalization of the government’s investment on health policies, programmes or projects. This investment will be returned by improved economic growth, and by lifting many out of poverty and maintaining the vibrancy and enhancement of many of the world’s largest cities.

Our study has several limitations stemming mainly from the limited reliable data available for many of the inputs. Data on intervention costs in low- and middle-income countries are particularly sparse. We believe the *Disease control priorities* cost information is the best currently available data, but many of the costs are based on very few studies and in some cases, based on similar interventions because no reliable cost studies were found. Improved studies of local intervention costs in multiple sites are needed to improve estimates,^48^ including the benefits that may come with economies of scale in urban areas.^49^ Data on current coverage levels for several interventions are lacking. In our study, we used global estimates for lower-middle income countries for coverage and populations in need of mental health interventions and rehabilitation services from *Disease control priorities* because country-specific data are missing for both countries. Reliable mortality data for Bangladesh are also missing.

Better population health is a profitable investment, resulting in increased productivity and economic stability.^50^ Expanding health expenditure increases productivity and years lived with good health, and the health sector is a source of employment at every level, raising the national GDP.^34^ Sufficient funds for expansion of coverage of health interventions may not be immediately available, but future economic growth, driven by cities, is justifying that Bangladesh and India should expand investments in urban health.

## References

[1] Children and urban development: past and present. New York: United Nations Children’s Fund; 2012. Available from: https://www.unicef.org/about/history/index_61883.html [cited 2018 Aug 30].

[2] Urbanization beyond municipal boundaries: nurturing metropolitan economies and connecting peri-urban areas in India. Directions in development. Washington, DC: World Bank; 2013.

[3] 2018 revision of world urbanization prospects. New York: United Nations Department of Economic and Social Affairs, Population Division; 2018. Available from: https://www.un.org/development/desa/publications/2018-revision-of-world-urbanization-prospects.html [cited 2018 June 13].

[4] Jha P, Laxminarayan R. Choosing health: an entitlement for all Indians. Toronto: Centre for Global Health Research; 2009.

[5] Malhotra V, Banzon EP, Gelband H, Chin B, Wu DC, Khetrapal S, et al. Development of urban health investments in South Asia: a review. Manila: Asian Development Bank; 2019.

[6] Wu DC, Banzon EP, Gelband H, Chin B, Malhotra V, Khetrapal S, et al. Urban health investments in India and Bangladesh: rationale and equity considerations. Manila: Asian Development Bank; 2019.

[7] Watkins DA, Jamison DT, Mills A, Atun R, Danforth K, Glassman A, et al. Universal health coverage and essential packages of care. In: Jamison DT, Gelband H, Horton S, Jha P, Laxminarayan R, Mock CN, et al., editors. Improving health and reducing poverty: disease control priorities. 3rd ed. Washington, DC: World Bank; 2018. pp. 44–65.

[8] Wu DC, Banzon E, Gelband H, B Chin, Malhotra V, Khetrapal S, et al. Supplementary webappendix: Health-care investments for the urban populations, Bangladesh and India [data repository]. London: figshare; 2019. Available from: https://figshare.com/articles/Urban_health_Webappendix_revised_FINAL_docx/8984159 [cited 2019 Sep 13].

[9] Population and housing census 2011. Bangladesh: Bangladesh Bureau of Statistics; 2011.

[10] Registrar General and Census Commissioner of India. Census of India 2011: population enumeration data (final population). New Delhi: Ministry of Home Affairs, Government of India; 2011.

[11] Office of the Registrar General. Medical certification of cause of death; 1990–2010. New Delhi: Ministry of Home Affairs; 2010.

[12] Jha P, Gajalakshmi V, Gupta PC, Kumar R, Mony P, Dhingra N, et al.; RGI-CGHR Prospective Study Collaborators. Prospective study of one million deaths in India: rationale, design, and validation results. PLoS Med. 2005 2;3(2):e18. 10.1371/journal.pmed.0030018 16354108 PMC1316066

[13] Horton S, Levin C. Cost–effectiveness of interventions for reproductive, maternal, newborn, and child health. In: Black R, Temmerman M, Laxminarayan R, Walker N, editors. Reproductive, maternal, newborn, and child health: disease control priorities. Volume 2 3rd ed. Washington, DC: World Bank; 2016 pp. 319–34. 10.1596/978-1-4648-0348-2_ch17

[14] Horton S, Gauvreau C. Cancer in low- and middle-income countries: an economic overview. In: Gelband H, Jha P, Sankaranarayanan R, Horton S, editors. Cancer: disease control priorities. Volume 3 3rd ed. Washington, DC: World Bank; 2015 pp. 263–80. 10.1596/978-1-4648-0349-9_ch16 26913333

[15] Levin C, Chisholm D. Cost–effectiveness and affordability of interventions, policies, and platforms for the prevention and treatment of mental, neurological and substance use disorders. In: Patel V, Chisholm D, Dua T, Laxminarayan R, Medina M, editors. Mental, neurological, and substance use disorders: disease control priorities. Volume 4 3rd ed. Washington, DC: World Bank; 2016 pp. 219–36. 10.1596/978-1-4648-0426-7_ch12 27227237

[16] Gaziano T, Suhrcke M, Brouwer E, Levin C, Nikolic I, Nugent R. Costs and cost–effectiveness of interventions and policies to prevent and treat cardiovascular and respiratory diseases. In: Prabhakaran D, Anand S, Gaziano T, Mbanya J, Wu Y, Nugent R, editors. Cardiovascular, respiratory, and related disorders: disease control priorities. Volume 5 3rd ed. Washington, DC: World Bank; 2017 pp. 349–67. 10.1596/978-1-4648-0518-9_ch19 30212069

[17] Holmes K, Bertozzi S, Bloom B, Jha P, Gelband H, De Maria LM, et al. Major infectious diseases: key messages from disease control priorities. In: Holmes K, Bertozzi S, Bloom B, Jha P, editors. Major infectious diseases: disease control priorities. Volume 6. 3rd ed. Washington, DC: World Bank; 2017. pp. 1–27. 30212102

[18] Watkins D, Dabestani N, Nugent R, Levin C. Interventions to prevent injuries and reduce environmental and occupational hazards: a review of economic evaluations from low- and middle-income countries. In: Mock CN, Nugent R, Kobusingye O, Smith K, editors. Injury prevention and environmental health: disease control priorities. Volume 7 3rd ed. Washington, DC: World Bank; 2017 pp. 199–211. 10.1596/978-1-4648-0522-6_ch10 30212115

[19] Reavley N, Patton G, Sawyer S, Kennedy E, Azzopardi P. Health and disease in adolescence. In: Bundy D, de Silva N, Horton S, Jamison DT, Patton G, editors. Child and adolescent health and development: disease control priorities. Volume 8. 3rd ed. Washington, DC: World Bank; 2017. pp. 239–52. 30212139

[20] Horton S, De la Cruz Toledo E, Mahon J, Santelli J, Waldfogel J. Identifying an essential package for adolescent health: economic analysis. In: Bundy D, de Silva N, Horton S, Jamison DT, Patton G, editors. Child and adolescent health and development: disease control priorities. Volume 8 3rd ed. Washington, DC: World Bank; 2017 pp. 369–84. 10.1596/978-1-4648-0423-6_ch26 30212133

[21] World development indicators. Washington, DC: World Bank; 2018. Available from: https://data.worldbank.org/indicator/PA.NUS.FCRF?locations=IN [cited 2018 Apr 30].

[22] Watkins DA, Qi J, Horton S. Costing universal health coverage: the DCP3 model. [working paper]. Seattle: University of Washington; 2017 Available from: http://dcp-3.org/sites/default/files/resources/20.%20Costs%20of%20UHC_Working%20Paper_Watkins%20_final%2013%20Nov_0.pdf [cited 2019 Sep 13].

[23] National Family Health Survey (NFHS-4), 2015–16: India. Mumbai and Rockville: International Institute for Population Sciences and ICF; 2017.

[24] Bangladesh Demographic and Health Survey 2014. Dhaka, Bangladesh, and Rockville. Rockville: National Institute of Population Research and Training, Mitra and Associates and ICF International; 2016.

[25] Bangladesh sample vital statistics 2016. Dhaka: Bangladesh Bureau of Statistics; 2017. Available from: http://bbs.portal.gov.bd/sites/default/files/files/bbs.portal.gov.bd/page/6a40a397_6ef7_48a3_80b3_78b8d1223e3f/SVRS_REPORT_2016.pdf. [cited 2018 May 5].

[26] Ferlay J, Soerjomataram I, Ervik M, Dikshit R, Eser S, Mathers C, et al. GLOBOCAN 2012 v1.0, Cancer Incidence and Mortality Worldwide: IARC CancerBase No. 11. Lyon: International Agency for Research on Cancer; 2013.

[27] National Centre of Disease Informatics and Research-National Cancer Registry Programme. Three-year report of the population based cancer registries 2012–2014. Bangalore: Indian Council of Medical Research; 2016.

[28] Global Burden of Disease Study 2015 (GBD 2015) reference life table. Seattle: Institute for Health Metrics and Evaluation; 2016.

[29] World malaria report 2016. Geneva: World Health Organization; 2016. Available from: https://apps.who.int/iris/bitstream/handle/10665/252038/9789241511711-eng.pdf?sequence=1 [cited 2018 Jun 04].

[30] Global tuberculosis report 2016. Geneva: World Health Organization; 2016. Available from: https://apps.who.int/medicinedocs/en/d/Js23098en/ [cited 2018 Jun 2].

[31] Watkins DA, Norheim OF, Jha P, Jamison D. Reducing mortality within universal health coverage: the DCP3 model. [working paper]. Seattle: University of Washington; 2017 Available from: http://dcp-3.org/resources/mortality-impact-achieving-essential-universal-health-coverage-low-and-middle-income [cited 2019 Sep 13].

[32] Norheim OF, Jha P, Admasu K, Godal T, Hum RJ, Kruk ME, et al. Avoiding 40% of the premature deaths in each country, 2010-30: review of national mortality trends to help quantify the UN sustainable development goal for health. Lancet. 2015 1 17;385(9964):239–52. 10.1016/S0140-6736(14)61591-9 25242039

[33] Jha P, Hum R, Gauvreau C, Jordan K. Benefits and Costs of the Health Targets for the Post-2015 Development Agenda. In: Lomborg B, editor. Prioritizing development: a cost benefit analysis of the United Nations’ sustainable development goals. Cambridge: Cambridge University Press; 2018 pp. 219–30. 10.1017/9781108233767.012

[34] Jamison DT, Jha P, Malhotra V, Verguet S. Human health: The twentieth century transformation of human health—its magnitude and value. In: Lomborg B, editor. How much have global problems cost the world? A scorecard from 1900. Cambridge: Cambridge University Press; 2013: 207–46. 10.1017/CBO9781139225793.009

[35] Disease burden and mortality estimates [internet]. Geneva: World Health Organization; 2018. Available from: https://www.who.int/healthinfo/global_burden_disease/estimates/en/index1.html [cited 2018 Jun 12].

[36] Pai M, Correa N, Mistry N, Jha P. Reducing global tuberculosis deaths-time for India to step up. Lancet. 2017 3 25;389(10075):1174–6. 10.1016/S0140-6736(17)30790-0 28353428

[37] National health profile. New Delhi: Ministry of Health and Family Welfare; 2018.

[38] Global health expenditure database [internet]. Geneva: World Health Organization; 2018. Available from: http://apps.who.int/nha/database [cited 2019 Jan 21].

[39] Lindert PH, editor. Growing Public: Social spending and economic growth since the eighteenth century. Volume 1 Cambridge: Cambridge University Press; 2004.

[40] Jowett M, Kutzin J. Raising revenues for health in support of UHC: strategic issues for policy makers. Health financing policy brief no. 1. Geneva: World Health Organization; 2015.

[41] Report of the National Commission on Macroeconomics and Health. New Delhi: Ministry of Health and Family Welfare; 2005.

[42] Status report on macroeconomics and health: Bangladesh. Geneva: World Health Organization; 2004. Available from: https://www.who.int/macrohealth/action/en/rep04_bangladesh.pdf. [cited 2019 Jul 23].

[43] Health systems: questions and answers on universal health coverage [internet]. Geneva: World Health Organization; 2018. Available from: https://www.who.int/healthsystems/topics/financing/uhc_qa/en/ [cited 2019 Jan 21].

[44] World health report 2010: health systems financing: the path to universal coverage. Geneva: World Health Organization; 2010. Available from: http://apps.who.int/medicinedocs/en/m/abstract/Js20169en/ [cited 2019 Nov 29]. 10.2471/BLT.10.078741 PMC2878164 20539847

[45] Gupta S. India's forex market maange more. Forbes India. 2017 Dec 6. Available from: http://www.forbesindia.com/blog/uncategorized/indias-forex-market-maange-more/ [cited 2018 Jan 10].

[46] Global Tobacco Economics Consortium. The health, poverty, and financial consequences of a cigarette price increase among 500 million male smokers in 13 middle income countries: compartmental model study. BMJ. 2018 4 11;361:k1162. 29643096 10.1136/bmj.k1162 PMC5894369

[47] Jamison DT, Alwan A, Mock CN, Nugent R, Watkins D, Adeyi O, et al. Universal health coverage and intersectoral action for health: key messages from disease control priorities, 3rd edition. Lancet. 2018 3 17;391(10125):1108–20. 10.1016/S0140-6736(17)32906-9 29179954 PMC5996988

[48] Horton S. Cost-effectiveness analysis in disease control priorities. In: Jamison DT, Gelband H, Horton S, Jha P, Laxminarayan R, Mock CN, et al., editors. Improving health and reducing poverty: disease control priorities. 3rd ed. Washington, DC: World Bank; 2018. pp. 147–56.

[49] Wu DC, Banzon EP, Gelband H, Chin B, Malhotra V, Khetrapal S, et al. Costs and benefits of essential urban health services in India and Bangladesh. Manila: Asian Development Bank; 2019.

[50] Alleyne G, Evans T, Jha P, Veillard J. Enhancing human capital and boosting productivity by tackling non-communicable diseases: A joint agenda for countries and partners. Washington, DC: World Bank; 2019.

